# Phylogeography of *Pulsatilla cernua* (Ranunculaceae), a grassland species, in Japan

**DOI:** 10.1002/ece3.5298

**Published:** 2019-06-13

**Authors:** Asuka Takaishi, Andrey E. Kozhevnikov, Zoya V. Kozhevnikova, Hajime Ikeda, Noriyuki Fujii, Akiko Soejima

**Affiliations:** ^1^ Department of Biological Science, Faculty of Advanced Science and Technology Kumamoto University Kumamoto Japan; ^2^ Federal Scientific Center of the East Asia Terrestrial Biodiversity Far Eastern Branch of the Russian Academy of Sciences Vladivostok Russia; ^3^ Institute of Plant Science and Resources Okayama University Kurashiki Japan

**Keywords:** continental‐grassland relict, East Asia, Japan, Mansen plant, phylogeography

## Abstract

The genetic diversity and structure of *Pulsatilla cernua,* a continental‐grassland relict, were investigated using variations in chloroplast DNA (cpDNA) and microsatellites of nuclear DNA. In the analyses of three cpDNA regions, 17 haplotypes were found in 24 populations of *P. cernua* from Japan, Korea, and Russia. Although the route and time of migration between the continent of Asia and Japan could not be well resolved, the cpDNA haplotype network suggests the existence of several ancient lineages in Japan and a recent secondary migration from Japan to the continent. Microsatellite analyses did not indicate genetic structure among the Japanese populations, indicating the existence of gene flow across the distribution area until recently. These results indicate that the present fragmentation of *P. cernua* in Japan may reflect a rapid, recent reduction from a previously large, continuous distribution.

## 1 INTRODUCTION

Recent phylogeographic studies have explained historical changes in species distribution based on the genetic structure of extant species (Avise, 2000, 2004 etc.). Global climatic oscillations during the Quaternary caused major changes in the distribution of many species (Hewitt, 2000, 2004; Leipold, Tausch, Poschlod, & Reisch, 2017; Listl, Poschlod, & Reisch, 2017; Tausch, Leipold, Poschlod, & Reisch, 2017). Even though East Asia was primarily free of ice sheets during the last glacial period (from approximately 2.6 million to 11,700 years ago), climatic oscillations during the Quaternary influenced the distribution of vegetation (Harrison, Yu, Takahara, & Prentice, 2001; Qiu, Fu, & Comes, 2011).

The Japanese Archipelago extends along a 3,000 km line from the northeast to the southwest, covering a wide range of climatic zones, from subarctic to subtropical. The distribution ranges of most plants in the Japanese Archipelago have repeatedly shifted following climatic changes during the Quaternary (Ikeda, Carlsen, Fujii, Brochmann, & Setoguchi, 2012; Kubota, Kusumoto, Shiono, & Tanaka, 2017; Yoshida, Kudo, Shimada, Hashizume, & Ono, 2016). Because their range shifts in the archipelago interacted with the neighboring continent of Asia as well as associated islands (Chiang et al., 2014; Fujii, Ueda, Watano, & Shimizu, 1997; Ikeda, Higashi, Yakubov, Barkalov, & Setoguchi, 2014; Lee, Lee, & Choi, 2013; Nakamura et al., 2014; Sakaguchi et al., 2012), elucidating the historical interaction with the continent and surrounding islands is necessary to understand the origin of Japanese flora. Thus, investigating the processes that have led to changes in their distribution may help us to understand their origin.

In the Japanese flora, there is a group of temperate grassland plants that are distributed in northeastern China, Far East Russia, the Korean Peninsula, and Japan. Koizumi (1931 ) named them “Mansen plants,” after the geographical names of the continental regions mentioned above (Man‐shu and Cho‐sen in Japanese). On the continent, they commonly occur in meadows and constitute temperate grassland vegetation that is widely spread across northeastern China. Their continental range is likely the place of origin of the Mansen plants (Murata, 1988; Tabata, 1997). In Japan, most of these plants occur in the temperate southwestern part of the archipelago and are not found on the northernmost large island of Hokkaido (Hotta, 1974; Koizumi, 1931; Murata, 1988). Based on their present distribution in Japan, it is hypothesized that these plants migrated to Japan via the Korean Peninsula (Hotta, 1974; Kitamura, 1957; Murata, 1988; Tabata, 1997). Their distribution areas would have been expanded under the cool and dry climate of the glacial age in Pleistocene, followed by reduction in the postglacial period. Ushimaru, Uchida, and Suka (2018) called these plants "continental‐grassland relicts." Kitamura (1957) considered that many of the Mansen plants immigrated to Japan approximately 150,000 years ago through the land bridge between the Korean Peninsula and Kyushu; however, he noted the possibility of much older immigration through a northern route of ancient land bridges in several species. Hotta (1974) also postulated multiple immigrations at different periods, based on the existence of various plant groups, such as warm temperate species (e.g., *Potentilla discolor* Bunge) and cool temperate species (e.g., *Ribes maximowiczianum* Kom.).

Today, natural temperate grasslands in Japan are restricted to places influenced by periodic disturbances, such as fires, floods, and volcanic eruptions. The outlines of their distribution at present occupy a rather large area in Japan, but most Mansen plants are found only in a few small isolated populations. Warming temperatures after the last glacial period probably caused habitat fragmentation due to encroaching forests (Suka, 2012; Tabata, 1997).

*Pulsatilla cernua* (Thunb.) Bercht. et C. Presl., a Mansen plant (Hotta, 1974; Murata, 1977, 1988), is a perennial herb that grows in the sunny grasslands of low mountains and river floodplains of Honshu, Shikoku, and Kyushu. This species has a relatively wide distribution in Japan, from Kyushu to the northern part of Honshu. They could have immigrated to Japan from the west and expanded eastward to the northern end of Honshu. In that case, we expect closer relationship between the western populations and continental ones. Otherwise, they might have moved into Japan from the north and expanded westward to Kyushu, followed by extinction in Hokkaido. To understand migration history of Mansen plants in Japan, the genetic diversity and structure among populations of *P. cernua* were investigated.

## 2 MATERIALS AND METHODS

### 2.1 Plant materials and DNA extraction

**Figure 1:**
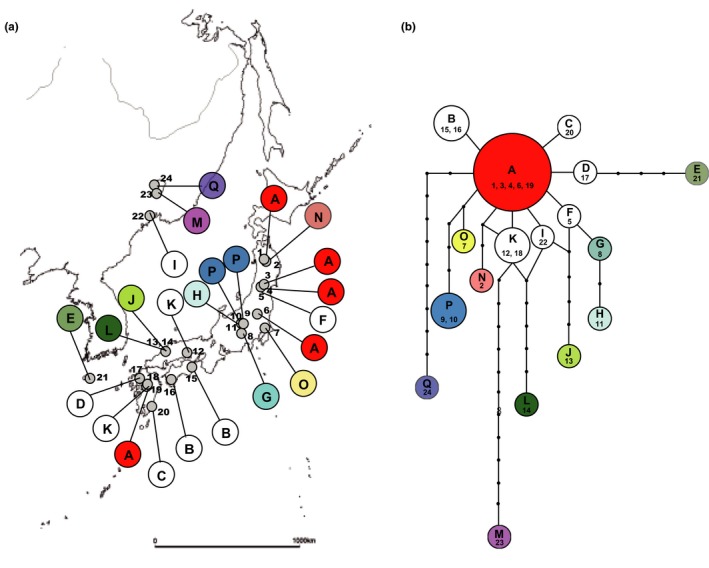
(a) Distribution of the cpDNA haplotypes of *Pulsatilla cernua*, (b) cpDNA haplotype network based on statistical parsimony. Circle size is proportional to the number of populations in the haplotype. Haplotypes with one‐step distance from haplotype A are colored with white

One hundred eighty‐nine individuals from 24 populations of *P. cernua* were collected in Japan, Korea, and Russia (Figure 1a, Table 1). The voucher specimens were deposited at Kumamoto University (KUMA). The leaves were dried in silica gel, and total genomic DNA was extracted using a modified cetyltrimethylammonium bromide (CTAB) method (Doyle & Doyle, 1987).

**Table 1:** Collection localities of *Pulsatilla cernua*

	Population no.	Location	Sample size	Haplotype	Voucher
Japan	1	Appi Plateau, Iwate Pref.	8	A	Soejima & Takaishi 131008
	2	Kamisodegawa, Iwate Pref.	8	N	Takaishi 160608
	3	Yamagata Airport, Yamagata Pref.	8	A	Soejima & Takaishi 140613‐1
	4	Obanazawa City, Yamagata Pref.	8	A	Soejima & Takaishi 140613‐2
	5	Shirataka Town, Yamagata Pref.	8	F	Soejima & Takaishi 140614
	6	Shioya Town., Tochigi Pref.	8	A	Takaishi 130421
	7	Inzai City, Chiba Pref.	8	O	Nishiihiro 130930
	8	Mt. Tennyo, Yamanashi Pref.	5	G	Soejima & Takaishi 140806
	9	Sugadaira, Nagano Pref.	8	P	Soejima & Takaishi 140807‐3
	10	Minegahara, Nagano Pref.	8	P	Soejima & Takaishi 140807‐1
	11	Dabos Hills, Nagano Pref.	8	H	Soejima & Takaishi 140807‐2
	12	Wake Town, Okayama Pref.	8	K	Soejima & Takaishi 150617
	13	Mt. Sanbe (west side), Shimane Pref.	8	J	Soejima & Takaishi 150618‐1
	14	Mt. Sanbe (north side), Shimane Pref.	8	L	Soejima & Takaishi 150618‐2
	15	Wajiki Town, Tokusima Pref.	8	B	Soejima 131101
	16	Shimanto City, Kochi Pref.	8	B	Soejima 130730
	17	Mt. Kizan, Saga Pref.	8	D	Takaishi 150404
	18	Aso City, Kumamoto Pref.	8	K	Soejima & Takaishi 130520
	19	Mt. Ichinomine, Kumamoto Pref.	8	A	Soejima & Takaishi 1304
	20	Cape Toi, Miyazaki Pref.	8	C	Soejima 130808
Korea	21	Cheju Island	8	E	Soejima & Takaishi 160616
Russia	22	Narva Bay, Primorsky Krai	8	I	Soejima & al. 150510
	23	Komissarovo, Primorsky Krai	8	M	Soejima & al. 150507
	24	Turiy Rog, Primorsky Krai	8	Q	Soejima & al. 150506

### 2.2 Chloroplast DNA sequencing and microsatellite genotyping

In a preliminary investigation, six noncoding regions of cpDNA were sequenced for eight populations using one plant from each population. These regions were a*tpB‐rbcL, trnH‐psbA, psbC‐trnS, trnD‐trnT, trnH‐trnK*, and *trnL‐trnF*. Based on the existence of intraspecific variation, three regions (*trnL‐trnF,* trnH‐*psbA,* and *trnH‐trnK*) were selected for further analysis (Table 2). No variation was found in the other three regions.

**Table 2:** Primers used for cpDNA amplification

Region	Primer	Sequence (5′–3′)	References
*trnL‐trnF*	trnLe	GGTTCAAGTCCCTCTATCCC	Taberlet, Gielly, Pautou, and Bouvet, (1991)
	trnFf	ATTTGAACTGGTGACACGAG	
*psbA‐trnH*	trnH (GUG)	ACTGCCTTGATCCACTTGGC	Hamilton, (1999)
	psbA	CGAAGCTCCATCTACAAATGG	
*trnH‐trnK*	trnH	ACGGGAATTGAACCCGCGCA	Demesure, Sodzi, and Petit, (1995)
	trnK	CCGACTAGTTCCGGGTTCGA	

PCR involved one cycle of 5 min at 94°C, followed by 35 cycles for 1 min at 94°C, 1 min at 52°C (*trnL‐trnF, trnH‐psbA*), or 1 min at 58°C (*trnH‐trnK*), and 3 min at 72°C, followed by 5 min at 72°C, using a DNA thermal cycler (Takara, Otsu, Japan). The PCR products were purified with the Illustra Enzymatic PCR and Sequencing Clean‐up Kit (GE Healthcare) to remove excess primers and dNTPs. Purified DNA fragments were used as templates for sequencing reactions, using the ABI Big Dye Terminator Cycle Sequencing Kit (Applied Biosystems), and were sequenced by an ABI automated sequencer. The obtained sequences were aligned using CLC Main Workbench 7.6.4 (Qiagen) and MEGA 6 (Tamura, Stecher, Peterson, Filipski, & Kumar, 2013), and they were manually adjusted where necessary. The simple indel coding method (Simmons & Ochoterena, 2000) was employed for gap coding.

To analyze microsatellite variation, 20 microsatellite loci, used for *P. patens* (Szczecińska, Kwaśniewski, Chwiałkowska, & Sawicki, 2013) and *P. vulgaris* (Dileo, Graf, Holderegger, Rico, & Wagner, 2015), were initially used for *P. cernua*. Among them, six loci (pul02, pul03, pul04, pul05, pul06, and PV65), which amplified constantly and were polymorphic, were chosen for further analysis (Table 3). The PCR cycles followed Szczecinska et al. (2013) and Dileo et al. (2015). Amplified fragment lengths were determined using CEQ 8,000 DNA Analysis System software (Beckman Coulter, Inc.).

**Table 3:** Primers used for amplification of microsatellite regions

Primer	Repeat motif	Sequence (5′–3′)	References
Pul02F	AC	TGAGTTCTTGCACTTCAGGG	Szczecinska et al. (2013)
Pul02R		AATCCCACGAGTTAGTGCC	
Pul03F	GAT	AGGTTGGAGGAAGCTTTAATGG	
Pul03R		TCCGGTGAACTCGAAGC	
Pul04F	CT	ACCGTTACTGTCCAACGGG	
Pul04R		CCTGTATGAATGCAACTTGACG	
Pul05F	CT	GATTAATGGCGGGCGACAG	
Pul05R		TGGGTGTCGCTAATCGAGG	
Pul06F	ATT	TGGCATTCCTAGTTGAGGATGG	
Pul06R		GCTAGACAAACAAGAATCCCTGC	
PV65f	AG	ACGGACGCAAATCTTCTGAC	Dielo et al. (2015)
PV65r		GAGAACGAACGCCATGAGAG	

### 2.3 Data analysis

#### 2.3.1 Haplotype network

The three noncoding cpDNA regions were subsequently concatenated and analyzed. The concatenated sequences were used to construct an unrooted haplotype network, including the 24 samples (one from each population) of *P. cernua* using statistical parsimony software TCS 1.21 (Clement, Posada, & Crandall, 2000). Insertions and deletions were all nonoverlapping and were included as single‐gap characters for statistical parsimony analysis.

#### 2.3.2 Genetic diversity

One hundred eighty‐nine samples (eight from each population, except for five from the Yamanashi population) were used for the microsatellite analyses. Although eight individuals per population is a comparatively low sample size to estimate genetic structure, the mean number of alleles (*N_A_*), total number of alleles (*T_A_*), observed (*H_O_*) and expected (*H_E_*) heterozygosities (Weir & Cockerham, 1984), and the fixation index (*F_IS_*) (Weir & Cockerham, 1984) were calculated using GenAlEx 6.5 (Peakall & Smouse, 2012) and averaged over all loci in each population. Linkage disequilibria were tested by the Markov chain algorithm for all pairwise combinations of the loci with sequential Bonferroni corrections (Rice, 1989) with GENEPOP version 4.0 (Rousset, 2008). Any significant deviation of *F_IS_* from zero was evaluated with 1,000 randomizations using FSTAT version 2.9.3.2 (Goudet, 1995).

#### 2.3.3 Population structure based on microsatellite data

To examine population structure, an individual‐based assignment approach was used, assuming correlated allele frequencies and admixed ancestry, and implemented in STRUCTURE 2.3 (Pritchard, Stephens, & Donnelly, 2000). The analysis assumes *K* clusters and assigns individuals to one or more clusters through Markov chain Monte Carlo (MCMC) simulation. STRUCTURE was run 10 times for *K* = 1 to *K* = 24 clusters, and the length of the burn‐in was set to 100,000, followed by 300,000 MCMC iterations. The most likely *K* value was determined by calculating *∆K* (Evanno, Regnaut, & Goudet, 2005). The *∆K* calculations were performed using the online version of STRUCTURE HARVESTER version 0.6.91 (Earl & von Holdt, 2012). To compare the likelihood between *K* = 1 and *K* = 2, genetic differentiation of haplotypes between the two groups, designated as *K* = 2, was estimated by the genetic differentiation among subpopulations (*F_st_*) using DnaSP v6 (Rozas et al., 2017).

To consider genetic relationships among eastern Japan (pops. 1–11), western Japan (pops. 12–20), Korea (pop. 21), and Russia (pops. 22–24), principal component analysis (PCA) was applied to microsatellite data. The significance of differences in genetic diversity between eastern Japan (pops. 1–11) and western Japan (pops. 12–20), and between Japan (pops. 1–20) and Russia (pops. 22–24) was evaluated by the *U* test. Korea was excluded from the *U* test because our data were restricted to one single population from Korea.

#### 2.3.4 Correlation between genetic distance and geographic distance

We calculated the correlation coefficient between genetic distance and geographic distance for all the populations based on microsatellite data. The correlation coefficient was also calculated for the populations excluding the Russian populations, to avoid the influence of geographic barrier that is formed by the Sea of Japan. Geographic distance was transformed to Ln(1+GGD)). The Mantel permutation procedure (Mantel, 1967) was adopted to test for isolation by distance using GenAlEx 6.5 (Peakal & Smouse, 2012).

#### 2.3.5 Correlation between genetic diversity and geographic latitude/longitude

We calculated the correlation coefficient between genetic diversity and geographic latitude/longitude for Japanese populations based on microsatellite data.

## 3 RESULTS

### 3.1 Chloroplast DNA sequencing analysis

The concatenated and aligned cpDNA sequence was 1,620 bp. Seventeen haplotypes were identified among the 24 individuals sampled from the 24 populations of *P. cernua*. Four haplotypes (A, B, K, and P) were found in multiple populations (Figure 1a). Figure 1b shows an unrooted haplotype network using statistical parsimony. Two haplotypes from Russia (M and Q) were genetically distant from those from Japan, and these two haplotypes, and another haplotype from Russia (I), did not form a cluster in the network.

### 3.2 Genetic diversity within and between populations

None of the linkage disequilibria among the six microsatellite loci were significant after the sequential Bonferroni correction in all populations examined (*p* > 0.05, data not shown). Thus, the six loci were sufficiently independent to apply Bayesian clustering using the admixture, instead of the linkage model (Falush, Stephens, & Pritchard, 2003). Deviations from the genotypic proportions were within the range expected under Hardy–Weinberg equilibrium in all populations. No significant genotypic disequilibrium was detected following the Bonferroni correction.

The *N_A_* across loci ranged from 1.2 to 2.1, the mean *H_O_* and *H_E_* ranged from 0.083 to 0.396 and 0.092 to 0.458, respectively, and the *F_IS_* ranged from −0.299 to 0.464 (Table 4). Populations 12 and 24 had three private alleles (which found in only one population), population 7 had two private alleles, and populations 9, 20, and 22 had one private allele. The range of genetic distances between the populations was 0.036–0.442 (Table 5). For the genetic diversity, *U* test indicated that there was no significant difference between the populations of eastern Japan and western Japan (Ho: *p* = 0.45, He: *p* = 1.00), nor between the populations of Japan and Russia (Ho: *p* = 0.493, He: *p* = 0.855).

**Table 4:** Sample number (*N*), mean number of alleles (*N_A_*), total number of allele (*T_A_*), observed heterozygosity (*H_O_*), expected heterozygosity (*H_E_*), and inbreeding coefficient (*F_IS_*) of 24 populations of *Pulsatilla cernua*

Population	*N*	*N_A_*	*T_A_*	*H_O_*	*H_E_*	*F_IS_*
1	8	1.4	12	0.083	0.193	0.425
2	8	1.9	17	0.298	0.391	0.237
3	8	1.6	13	0.208	0.305	0.404
4	8	1.5	14	0.327	0.281	−0.115
5	8	1.9	15	0.333	0.301	−0.167
6	8	1.6	15	0.194	0.252	0.183
7	8	1.5	14	0.104	0.227	0.464
8	5	1.2	9	0.200	0.140	−0.296
9	8	1.4	12	0.125	0.208	0.194
10	8	1.5	11	0.188	0.230	0.186
11	8	1.3	11	0.134	0.177	0.105
12	8	1.7	17	0.313	0.363	0.157
13	8	1.5	12	0.229	0.242	0.014
14	8	1.4	11	0.271	0.241	0.032
15	8	1.2	8	0.083	0.092	0.099
16	8	1.6	15	0.253	0.323	0.239
17	8	1.6	12	0.396	0.301	−0.299
18	8	1.3	10	0.188	0.163	−0.075
19	8	1.2	12	0.125	0.147	0.165
20	8	1.5	13	0.250	0.267	−0.004
21	8	2.1	16	0.292	0.458	0.383
22	8	1.9	15	0.271	0.430	0.388
23	8	1.4	12	0.292	0.216	−0.270
24	8	1.3	13	0.193	0.188	−0.064
Average		1.52	12.9	0.223	0.256	0.099

**Table 5:** Genetic distance (*F_st_*) between populations

Population	1	2	3	4	5	6	7	8	9	10	11	12	13	14	15	16	17	18	19	20	21	22	23
1																							
2	0.141																						
3	0.146	0.099																					
4	0.136	0.061	0.047																				
5	0.166	0.082	0.034	0.036																			
6	0.077	0.080	0.125	0.094	0.116																		
7	0.100	0.096	0.088	0.100	0.091	0.052																	
8	0.147	0.102	0.151	0.143	0.121	0.073	0.057																
9	0.132	0.097	0.101	0.101	0.101	0.069	0.068	0.070															
10	0.212	0.061	0.100	0.069	0.085	0.106	0.100	0.106	0.066														
11	0.167	0.134	0.089	0.089	0.094	0.116	0.098	0.129	0.049	0.088													
12	0.125	0.142	0.143	0.140	0.153	0.089	0.052	0.138	0.104	0.202	0.136												
13	0.200	0.062	0.114	0.116	0.113	0.128	0.075	0.138	0.126	0.119	0.175	0.150											
14	0.122	0.088	0.098	0.076	0.112	0.122	0.132	0.191	0.143	0.157	0.173	0.160	0.129										
15	0.139	0.119	0.045	0.071	0.063	0.126	0.087	0.184	0.129	0.163	0.139	0.134	0.135	0.081									
16	0.185	0.144	0.070	0.090	0.077	0.201	0.155	0.251	0.203	0.190	0.222	0.231	0.205	0.080	0.050								
17	0.153	0.116	0.128	0.122	0.153	0.110	0.098	0.180	0.141	0.201	0.155	0.152	0.105	0.069	0.097	0.140							
18	0.286	0.088	0.215	0.188	0.231	0.106	0.074	0.138	0.129	0.178	0.188	0.216	0.078	0.206	0.259	0.343	0.178						
19	0.202	0.113	0.157	0.165	0.156	0.093	0.048	0.101	0.099	0.139	0.166	0.115	0.056	0.206	0.182	0.303	0.160	0.048					
20	0.141	0.040	0.162	0.123	0.154	0.052	0.075	0.077	0.095	0.091	0.158	0.126	0.078	0.152	0.187	0.278	0.125	0.045	0.070				
21	0.311	0.047	0.179	0.164	0.178	0.227	0.203	0.247	0.250	0.188	0.305	0.339	0.086	0.152	0.229	0.230	0.191	0.203	0.231	0.129			
22	0.137	0.109	0.091	0.092	0.116	0.129	0.137	0.194	0.123	0.146	0.110	0.177	0.122	0.081	0.107	0.131	0.104	0.213	0.183	0.155	0.147		
23	0.291	0.214	0.163	0.223	0.217	0.220	0.215	0.316	0.214	0.379	0.217	0.332	0.233	0.161	0.164	0.229	0.170	0.442	0.286	0.299	0.332	0.131	
24	0.150	0.144	0.108	0.102	0.145	0.138	0.145	0.225	0.140	0.242	0.178	0.169	0.184	0.058	0.081	0.112	0.117	0.306	0.222	0.212	0.274	0.113	0.167

### 3.3 Population structure

**Figure 2:**
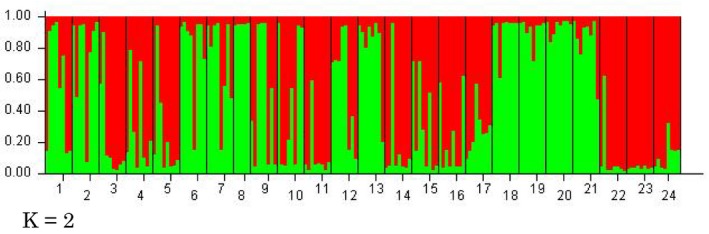
A result of Bayesian clustering in the STRUCTURE analysis. Bar plots show the probability of distinct clusters represented by two colors. The numbers on the horizontal axis indicate the populations shown in Table 1 and Figure 1a

STRUCTURE analyses showed that the most likely number of clusters using Bayesian cluster analysis was *K* = 2. Based on *K* = 2, we recognized two groups: red and green (Figure 2). To elucidate the main genetic structure, individuals that were assigned to a single cluster with more than 80% similarity were defined as red or green groups. However, even when analyzing these representative individuals, genetic differentiation between the red and the green groups, based on the haplotype sequences, was not significant (*F_st_* = 0.039, *p* = 0.449).

**Figure 3:**
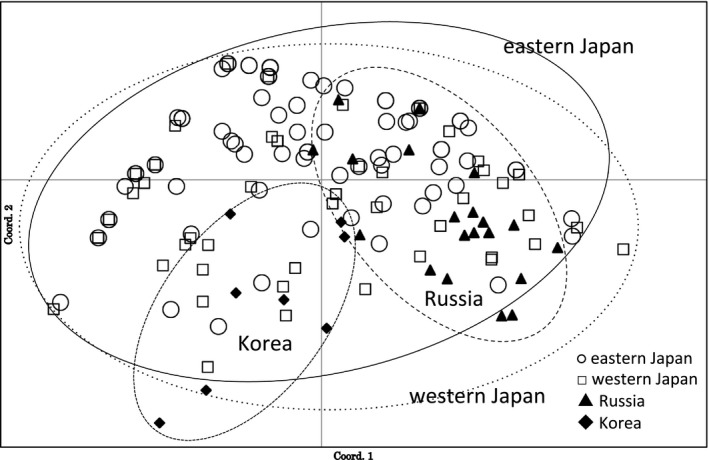
Scatter plot of scores of principal component 1 versus 2 from PCA for microsatellite data set. Each plot represents one plant. Plots of the same region, eastern Japan, western Japan, Russia, and Korea, were circumscribed.

Figure 3 shows the result of PCA based on the microsatellite data set. Each symbol represents a single plant. In this figure, the first two components explain 37.42% of the variance within the data set, with component 1 accounting for 19.97% and component 2 accounting for 17.45%. Ranges of genetic divergence of eastern Japan and western Japan are mostly overlapped. It seems that the ranges of Korean population and the Russian populations are separated, but they are included in the range of Japanese populations.

### 3.4 Correlation between genetic distance and geographic distance

**Figure 4:**
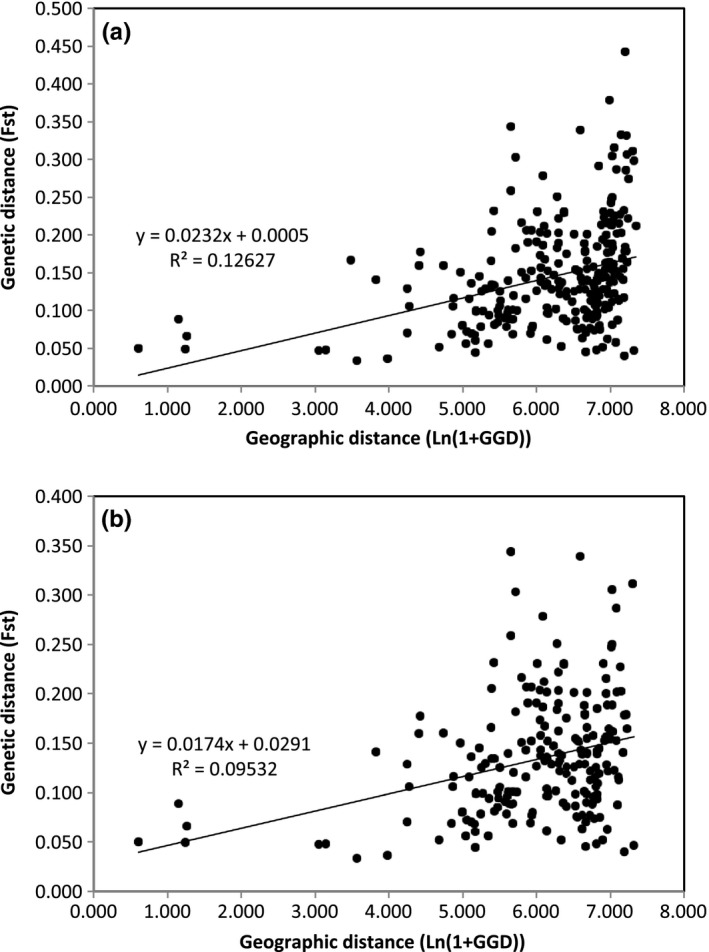
Correlation between geographic distance and genetic distance. (a): All the populations (*p* = 0.0001), (b): without Russian populations (*p* = 0.0001)

Figure 4 shows the relationship between the genetic distance and geographic distance based on the microsatellite data. The genetic distance (*F_st_*) between populations is ranged from 0.040 to 0.442 with an average 0.158 (Table 5). There was a positive correlation between geographical and genetic distance across populations, including or excluding Russian populations (Figure 4a,b). The correlation coefficient for all the populations was *R* ^2^ = 0.126 (*p* = 0.0001) and for the populations excluding the Russian populations was *R* ^2^ = 0.095 (*p* = 0.0001).

### 3.5 Correlation between genetic diversity and geographic latitude/longitude

**Figure 5:**
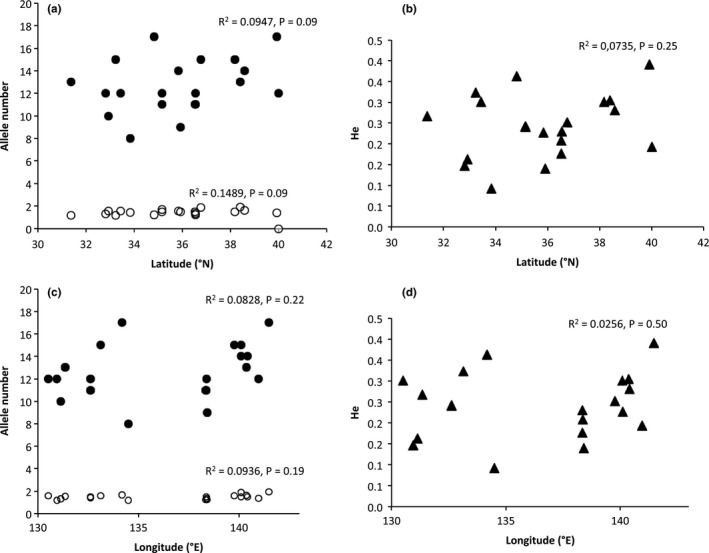
Correlation between genetic diversity and geographic latitude/longitude of Japanese populations. (a): Latitude and allele number (*N_A_* and *T_A_*), (b): latitude and H_E_, (c): longitude and allele number (*N_A_* and *T_A_*), (d): longitude and *N_E_*. Open circles indicate *N_A_*, close circles indicate *T_A_*, and close triangles indicate *H_E_*

Figure 5 shows the relationship between the genetic diversity and geographic latitude/longitude for Japanese populations based on the microsatellite data. There found weak correlation in each combination: between latitude and *N_A_* (*R* ^2^ = 0.1489, *p* = 0.09), latitude and *T_A_* (*R* ^2^ = 0.0947, *p* = 0.09), latitude and *H_E_* (*R* ^2^ = 0.0735, *p* = 0.25), longitude and *N_A_* (*R* ^2^ = 0.0936), longitude and *T_A_* (*R* ^2^ = 0.0828), and longitude and *H_E_* (*R* ^2^ = 0.0526, *p* = 0.50). However, all of the correlations were not significant.

## 4 DISCUSSION

### 4.1 Genetic diversity and genetic structure

The mean *N_A_* was 1.52 (range: 1.2–2.1), and the mean *H_E_* was 0.256 (range: 0.092–0.458) for the six microsatellite loci from the 24 populations (Table 4). In comparison, another congeneric species, *P. patens* (L.) Mill., a widely distributed circumboreal species, exhibited higher genetic diversity: *N_A_* = 3.74 and *H_E_* = 0.541 (Szczecińska et al., 2013). The diversity of another central European species, *P. vulgaris* Mill., was much higher: *N_A_* = 12.3 *H_E_* = 0.727 (Dileo et al., 2015). For Japanese grassland species, some previous studies reported *N_A_* = 12.0 and *H_E_* = 0.840 for *Silene kiusiana* (Makino) H. Ohashi & H. Nakai (Caryophyllaceae) (Yamasaki et al., 2013), and *N_A_* = 4.7 and *H_E_* = 0.791 for *Adenophora palustris* Kom. (Campanulaceae) (Masumoto, Kaneko, Otake, & Isagi, 2011). Although *P. cernua* is currently classified as vulnerable (VU) in the Japanese Red Data Book (Ministry of the Environment, 2015), it still has a wider distribution area than *S. kiusiana* (northern Korean Peninsula and western Japan) and larger populations than *A. palustris* (known only from a few small populations in western Japan). In *P. cernua*, we found more than a few hundred plants in several populations, but they had lower genetic diversity than the other abovementioned species: populations 3 (*N_A_* = 1.6, *H_E_* = 0.305), 7 (*N_A_* = 1.5, *H_E_* = 0.227), and 9 (*N_A_* = 1.4, *H_E_* = 0.208). Although population size is very important to consider genetic diversity, data on population size were not available in this study.

For genetic structure among the populations, STRUCTURE analyses based on microsatellite indicated *K* = 2. However, there was no significant difference between the two groups based on the haplotype sequences (*F_st_* = 0.039, *p* = 0.449). The result of PCA analysis on microsatellite also did not support two groups (Figure 3). On the other hand, the genetic distance is correlated with the geographic distance (Figure 4). These results mean low genetic differentiation between populations and suggest that the present fragmentation of *P. cernua* may reflect a rapid, recent reduction from a large, continuous distribution. It is noted that *P. cernua* was a common species in Japan before the era of high economic growth (Murata, 1988; Suka, 2012). During the 1960s, Japanese grasslands declined rapidly. Nakahama, Uchida, Ushimaru, and Isagi (2018) determined that the recent decline in the genetic diversity and effective population size of *Melitaea ambigua,* a grassland butterfly species, between the 1980s and the 2010s was due to the rapid loss of seminatural grasslands. The rapid loss of seminatural grasslands has also resulted in the significant reduction of genetic diversity in *P. cernua*.

### 4.2 Genetic relationship among populations

Seventeen haplotypes of cpDNA were found in the 24 populations of *P. cernua* (Figure 1). Among these haplotypes, just four (A, B, K, and P) occurred in multiple populations. Haplotype A was widely distributed in Tohoku, Kanto, and Kyusyu. Haplotypes B, K, and P were found in two populations each. The two populations with B and the two with P were closely located, whereas haplotype K was found in two disjunct populations: Okayama and Kumamoto (Figure 1a).

Haplotype A was located at the center of the haplotype network, while the other Japanese haplotypes were radially arranged, with distances of 1–7 mutations (Figure 1b). It seems that derivative radiation occurred in different regions after haplotype A became widely distributed in Japan. Because the distances between haplotype A and most of the other derivative haplotypes were not large, these haplotypes probably diversified rather recently. However, haplotypes J and L had larger genetic distances from haplotype A than the other haplotypes. These populations probably became isolated from haplotype A before the other populations. It is possible that the common ancestor of haplotypes A, J, and L might have been diversified before the expansion of haplotype A. It should be noted that the genetic distance between J and L is wide, although their geographic distance is small. The small genetic distance between these populations (*F_st_* = 0.05) indicates gene flow between them. It may suggest haplotype polymorphism within a population.

In Russia, three haplotypes (I, M, and Q) were found in three populations of Primorsky, but they were distantly related to each other. Although these haplotypes were located at derived positions from haplotype A in the network (Figure 1b), the extreme distances between haplotypes A, M, and Q may indicate the existence of unknown common ancestors, rather than the ancestral position of haplotype A. The population with haplotype Q (pop. 24) possessed three private alleles in microsatellite analysis, suggesting ancient isolation of the population. The large genetic distances between these three haplotypes indicate the existence of genetic polymorphism before the occurrence of genetic radiation in Japan. Although the relationship between these haplotypes is not well resolved, it is interesting that haplotype I, which was found on a sand dune on the Russian coast of the Sea of Japan near Vladivostok, seems to have derived from haplotype A rather recently.

Haplotype E, recorded from the population of Cheju Island, Korea, has a linear relationship with haplotypes A and D in the network (Figure 1b). It could have derived from haplotype A, or may possibly be an ancestor of haplotype A.

### 4.3 Migration and history of *P. cernua*, a Mansen plant

In general, the Mansen plants, namely continental‐grassland relicts in Japan, are considered to have originated in the temperate grasslands on the continent and migrated to Japan, eastward via the Korean Peninsula, under the cold and dry climate of the glacial age (Hotta, 1974; Koidzumi, 1931; Murata, 1988). The haplotype network shows that the widely distributed Japanese haplotype A is connected to the Korean haplotype E, by the intermediary haplotype D in Kyushu (Figure 1b). This concurs with the abovementioned western‐route hypothesis. But it is not sure because only a few populations from the continent were included in this study. Also, an immigration route from the north is not denied yet. The genetic diversity of the populations of northeastern Japan is slightly higher than that of western Japan (Figure 5). Although the correlation is not significant, it is possible that the higher northeastern genetic diversity reflects their older age.

In Japan, the large distribution area of haplotype A and the existence of its satellite haplotypes (haplotypes B, C, D, F, G, H, I, K, N, and O) imply that the rapid expansion of haplotype A happened rather recently, followed by diversification of the satellites. It is congruent with the consideration that grassland expanded on the Japanese archipelago most largely in the last glacier period (Suka, 2012; Tabata, 1997). In addition to the satellite haplotypes of the haplotype A, it is noted that the haplotypes J and L, found near the Sea of Japan, are genetically distant from other haplotypes. It is possible that there was an unknown common ancestor of the haplotypes A, J, and L before the expansion of haplotype A. On the other hand, the occurrence of haplotype I, closely related to haplotype A, in Russia suggests a recent migration from Japan to the continent. Although the genetic distance between the haplotypes of Japan and continent, microsatellite analysis did not indicate genetic differentiation among them (Figure 3). These results suggest a relatively long and complicated migration history for *P. cernua* in Japan, so that multiple distribution range shifts before the last glacial period must be taken into consideration. To elucidate the migration route and the range fluctuation of *P. cernua*, a Mansen plant, it is necessary to do further investigation using more genetic markers and samples from the continent.

## CONFLICT OF INTEREST

None declared.

## AUTHOR CONTRIBUTIONS

A.S. conceived the ideas; A.T., A.E.K, Z.V.K., N.F., and A.S. made fieldworks and collected the data; and A.S., A.T., and H.I. analyzed the data; and A.S. led the writing.

## Data Availability

All chloroplast sequences and microsatellite genotypes are submitted to the Dryad database on 13 May 2019. https://doi.org/10.5061/dryad.3mf353m.
